# Assessment of SMA Electrical Resistance Change during Cyclic Stretching with Small Elongation

**DOI:** 10.3390/s21206804

**Published:** 2021-10-13

**Authors:** Sebastian Sławski, Marek Kciuk, Wojciech Klein

**Affiliations:** 1 Department of Theoretical and Applied Mechanics, Silesian University of Technology, Konarskiego 18A, 44-100 Gliwice, Poland; wojciech.klein@polsl.pl; 2 Department of Mechatronics, Silesian University of Technology, Akademicka 2A, 44-100 Gliwice, Poland; marek.kciuk@polsl.pl

**Keywords:** shape memory alloy, cyclic testing, sensor, resistance, stretching, Flexinol, control algorithm

## Abstract

In this article, changes in NiTi alloy (Flexinol) electrical resistance during cyclic stretching with small elongation were investigated. A dedicated test stand consisting of motorized vertical test stand, force gauge, and electric resistance measuring device with an accuracy of 0.006 Ω was developed. A dedicated control algorithm was developed using LabVIEW software. Changes in electrical resistance were investigated for the 0.1 mm Flexinol wire with length of 120 mm. Testing was performed in the elongation range between 0.25% and 1.5% in martensite phase. Tested samples were subjected to 30 stretching cycles with a movement speed of 10 mm/min. Obtained results show that the cyclic stretching of Flexinol wire reduces its electrical resistance with each stretching cycle. Moreover, it was noted that changes in Flexinol electrical resistance during cycling stretching depend on the assumed elongation and number of the already performed stretching cycles. The observed electrical resistance change decreases with each stretching cycle. Thus, the observed changes are greater during the first stretching cycles. For elongations exceeding 1%, the Flexinol electrical resistance in the first stretching cycle increases. In each subsequent cycle, electrical resistance decreases, as in the case of the smallest value of assumed elongation. In almost all tested cases (except in the case with 1.5% of assumed elongation), Flexinol electrical resistance after 30 stretching cycles was smaller than before the test.

## 1. Introduction

Smart materials such as shape memory alloys (SMAs) are becoming increasingly popular in many industries. Due to changes in shape and mechanical properties accompanying the phase transformation of martensite into austenite, SMAs are often used as actuators. Sensors are another popular SMA application. This is due to the material property changes that occur during phase transformation. SMAs were discovered in 1932, making them the oldest example of this group, but their first applications were introduced in the 1960s when NiTi alloys (Nitinol) were invented [1]. Based on the chemical composition of the alloy, shape changes in SMAs are controlled by temperature. The shape changing mechanism is governed by martensitic transformation where the material phase changes from the martensite phase to the austenite phase. Nowadays, there are many SMA applications throughout a wide range of branches, including civil engineering as stress dampers, vibration control of cable stayed bridges, self-healing concrete, in space and aircraft as actuation systems, microgrippers, in medicine as orthodontic wires, in devices for the passive mobilization of bedridden patients, and pseudoelastic orthoses [2,3,4,5,6,7,8,9]. New SMAs are consistently being introduced and are the subject of countless reported studies. Phase changes can be described using different mathematical models based on mechanical or thermomechanical phenomena [10,11]. Simpler abstract models without physical background have also been proposed for hysteresis modelling as well [12]. A neural network for SMA hysteresis behavior modelling has also been applied [13,14]. In fact, three types of phases are observed for different thermal and mechanical combinations. The phases are the austenite, twinned martensite, and detwinned martensite. Changes of the SMA phase have an impact on the properties of the alloy including resistivity (ρ_o_). As a result, electrical resistance can be used for phase or displacement monitoring [15,16,17] in addition to feedback loops in SMA actuators. Passive SMA actuators are used in civil engineering for building protection against natural- or human-based vibrations, e.g., an earthquake or traffic [18].

**Figure 1:**
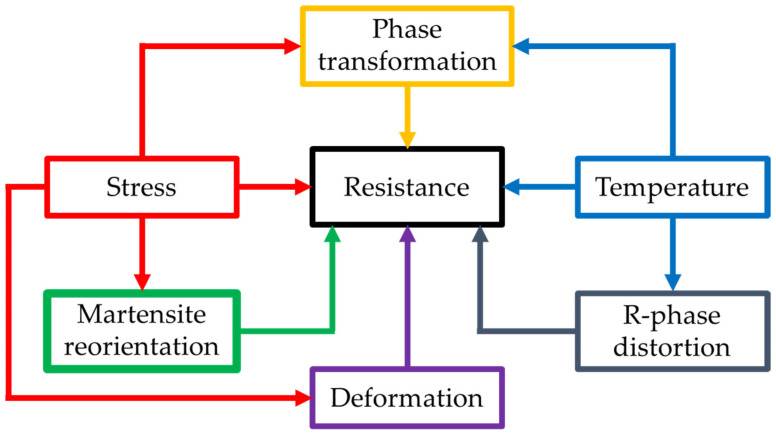
Factors influencing electrical resistance in SMA wires.

A second group of SMA applications comprise sensors based on thermally or mechanically induced electrical resistance changes. Liu et al. [19] first observed that the high sensitivity of SMA wires to strain made them strong candidates for sensor applications. Rączka et al. [20] reported that dynamic SMA resistance measurements correspond to phase transitions. Electrical resistance measurements could also be used as feedback in positioning systems based on thin SMA wires. However, electrical resistance in SMA wires is complex due to dynamic changes under the influence of factors such as temperature, r-phase distortion, stress, deformation, phase transformation, and martensite reorientation [21]. A scheme of electrical resistance influencing factors is presented in Figure 1.

R-phase distortion and phase transformation impacts on SMA resistance are caused by temperature change. This is because the phase changes that occur with temperature change in SMAs (i.e., from martensite (low temperature) to austenite (high temperature)). NiTi shape memory alloys transform martensitically from B2 cubic austenite into monoclinic B19′ martensite either directly or via rhombohedral R-phase [22]. R-phase is characterized by a narrow hysteresis loop, deformation level of about 1%, and fast response. One of the biggest challenge to utilize R-phase is functional and thermal stability [23]. The R-phase transformation commonly appears as an intermediate phase in most of the commercially available NiTi wires. The R-phase related deformation processes affect the overall thermomechanical behaviors of such wires [22]. The R-phase processes are least explored among all deformation/transformation processes in NiTi and their activity is very difficult to detect due to the small crystallographic strain associated with the R-phase and further peculiar features [24]. Šittner et al. [24] successfully used electric resistance measurements to investigate the thermomechanical behavior of the R-phase in NiTi wires. The electrical resistivity of SMAs during phase transformation are also dependent on applied stress [21]. Uchil et al. [25] investigated the relationship between electrical resistivity in SMAs with thermal cycles under constant tensile stress. The electrical resistivity was found to be dependent on the number of cycles. Electrical resistance in SMA wires also changes due to deformation. This is related to cross-sectional changes during stretching. As shown in Equation (1), a decreasing cross-sectional area increases the electrical resistance.

(1) $$R=\rho \cdot \frac{l}{A},$$

where *R* is the electrical resistance (Ω), ρ is the resistivity (Ω·m), l is the length (m), and A is the cross-sectional area (m^2^).

The martensitic SMA phase encompasses both twinned and detwinned martensite phases. Twinned martensite will form detwinned martensite during mechanical deformation, a process referred to as martensite reorientation [1,3]. This process affects the SMA electrical resistance and is permanent in low temperature phases. Due to martensite reorientation, SMAs can be used as online, offline, or combination sensors. Temperature phase transformation can be used to reset the sensor. SMA wire sensors offer an alternative to the currently used diagnostic solutions [26,27,28,29], e.g., as passive sensors which could provide information about too much deformation, stress in civil engineering, or modern ecological power supplies such as wind power plants.

SMA wire electrical resistance changes depending on various types of thermal and mechanical excitations. Wu et al. [30] examined NiTi shape memory alloy wire resistance during the thermo-mechanical loading and found both thermal and stress have an impact on electrical resistance change. Antonucci et al. [31] investigated dynamic SMA electrical resistivity during heating and cooling. The results showed electrical resistivity measurements to be a good probe for phase identification in SMAs. Novák et al. [32] investigated thermal and mechanical effects on electrical resistivity in NiTi wires showing R-phase transformation where the electrical resistivity of the martensite phase strongly increased with martensite reorientation under tension. Urbina et al. [33] measured the resistance of two-way shape memory NiTi wires during heating and cooling and observed that the ER measurements for trained samples show on cooling a decrease in resistivity that marks the transformation from R-phase to martensite. Li et al. [34] proposed a new repair method for simple reinforced concrete (RC) beams strengthened with carbon fiber reinforced polymer (CFRP) plates in combination with SMA wires based on the relationship between deflection and the change rate of the electric resistance. This kind of capability offers self-diagnosis and self-repairing functionality for SMAs towards the development of smart structures. Furs et al. noticed that the linearity and repeatability of the stress-resistance curve, particularly at high prestresses, make these materials ideal for applications that endeavor to use resistance measurements as a position feedback sensor [35]. Furs et al. [36] also noticed that the resistance–strain behavior that provides the basis for self-sensing in a system consisting of two opposing SMA wires and a spring flexure developed additional hysteresis compared to a system consisting of a single SMA in series with a linear spring.

A different approach is to use high current which induces the martensite–austenite transformation and causes contraction of the SMA, which works as both a sensor and actuator [37,38]. This system opens an electrical circuit. As long as the actuator is contracted, the SMA cools, and the austenite–martensite transformation occurs. In that measurement, the circuit supply voltage is observed.

Presented literature review shows that, the SMA have a lot of interesting application as actuators and sensors. Research concerning on SMAs are interest to many scientists from around the world. In this article, NiTi based Flexinol electrical resistance change during cyclic stretching with small elongation in martensite phase is investigated. This test refers to Flexinol electrical resistance changes caused by martensite reorientation from twinned to detwinned. This kind of study has not been presented in the literature. However, due to properties of the NiTi alloys, it could represent a valuable research direction which can potentially be used in Flexinol wire-based sensors. A potential application of such sensor is the monitoring of multilayered composite structures. Flexinol wires could be located between reinforced layers, inserted between the fibers, or applied as independent sensors glued to the diagnosed structure. Such sensors could measure, e.g., that the structure has not undergone too much deformation or count the number of deformations (material fatigue). Such information may be useful during the maintenance. Sensor based on Flexinol wires could work offline because changes in martensite (twinned to detwinned) are permanent until Flexinol is heated and cooled (restart by passing through the austenite phase). Resistance measurement could provide information concerning the need for detailed inspection or the replacement of a given composite component for a new one. Such a sensor could be particularly useful for large components made of multilayered composites materials, because their inspection is time-consuming and damage is often hard to detect. An example of such damage could be the collision of hail or other blunt elements into the blade of a wind turbine or a piece of the plane’s skin. The resulting damage could be hard to detect (particularly when it will be limited to delamination) and cause a significant decrease in the strength of such material. Another example could be the detection of too much deformation of the wind turbine blade under the influence of strong winds. In both mentioned cases, a Flexinol wire based sensor would allow a quick diagnosis of the composite structure. This article contains a description of the methodology and dedicated test stand development. The developed control algorithm (using LabVIEW) is also described. Results obtained during the research are presented in graphs and described. Observed changes in Flexinol electrical resistance during cycling stretching with small elongation are discussed. The article is summarized with the conclusions.

## 2. Materials and Methods

### 2.1. Methodology

**Figure 2:**
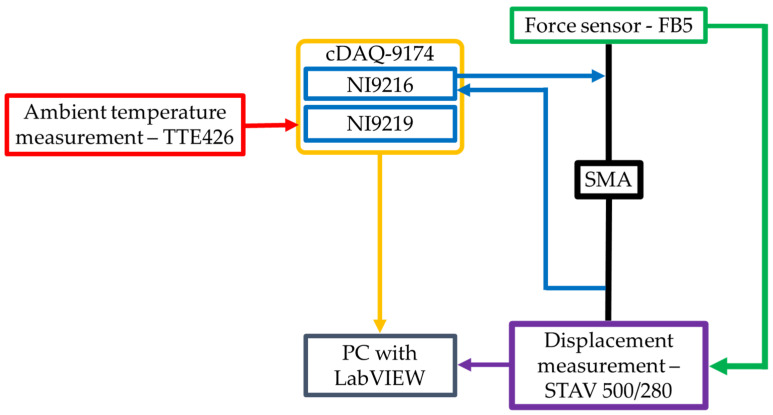
Schematic of testing stand.

Tests were conducted using a 120 mm length piece of 0.1 mm diameter wire made of NiTi alloy with the commercial name Flexinol [39]. This sample was tested using the developed test stand and the scheme is presented in Figure 2.

The testing stand was based on a STAV 500/280 (AXIS Sp. z o.o., Gdańsk, Poland) motorized vertical test stand with FB5 force gauge (AXIS Sp. z o.o., Gdańsk, Poland) and the cDAQ-9174 (NI, Austin, TX, USA) modular data acquisition system with NI9216 (NI, Austin, TX, USA) analog 24-bit for resistance measurements. The second module NI9219 (NI, Austin, TX, USA) targeted ambient temperature measurements. The STAV 500/280 communicated using a control unit via USB virtual serial port and the cDAQ communicated via USB using a dedicated transmission protocol. The NI 9216 was configured for a 4-wire resistance measurement using 1 mA stimuli current. The 4-wire method is preferred for resistance values below 100 Ω, and the specimen resistance value fell between 11 and 12 Ω. The low stimuli current does not increase the specimen temperature according to Joule–Lenz law, which is important according to the thermal behavior of SMA. The system controller is based on PC and works under a dedicated application written in LabVIEW 2019 programming environment (described in Section 2.2).

**Figure 3:**
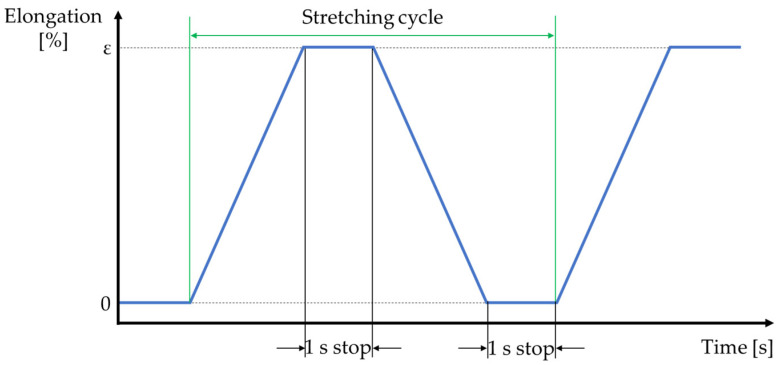
Schematic of the stretching cycle.

Flexinol wire samples were cyclically stretched by mounting the samples between two fixed and moving handles made from PLA. Various elongations ε were used as follows: 0.25% (0.3 mm), 0.5% (0.6 mm), 1% (1.2 mm), 1.25% (1.5 mm), and 1.5% (1.8 mm). The assumed stretching cycle relied on Flexinol wire stretching with a constant speed of 10 mm/min. Tested samples were stretched to the specified elongation then stopped for 1 s. A return to the initial position took place at the same speed (10 mm/min), and the pause between stretching cycles was also 1 s. A schematic of the assumed stretching cycle is presented in Figure 3.

Testing for each assumed elongation was performed twice. Before each test, specimens were restored to their natural shape using a heating-cooling sequence without a mechanical load. Afterwards, the moving handle was set at the start position. Each test consisted of 30 stretching cycles. Ambient temperature during the test was between 22 and 24 °C. Ambient temperature changes did not change the resistance of the tested sample to any noticeable degree. During the test, the displacement of the moving handle, stretching force, and sample resistance were measured with a frequency of 10 Hz. Displacement was measured by measuring system built into the motorized vertical test stand (STAV 500/280) used in the research. Stretching force was measured by a STAV 500/280 force gauge FB5 (with an accuracy of 0.005 N). The force gauge was mounted on the moving handle of the STAV 500/280. The measuring part of the force gauge was connected with the tested samples by a 3D-printed handle made of PLA. Measured force and displacement were sent to the PC with a control algorithm implemented in LabVIEW through the USB port.

### 2.2. Control Algorithm

**Figure 4:**
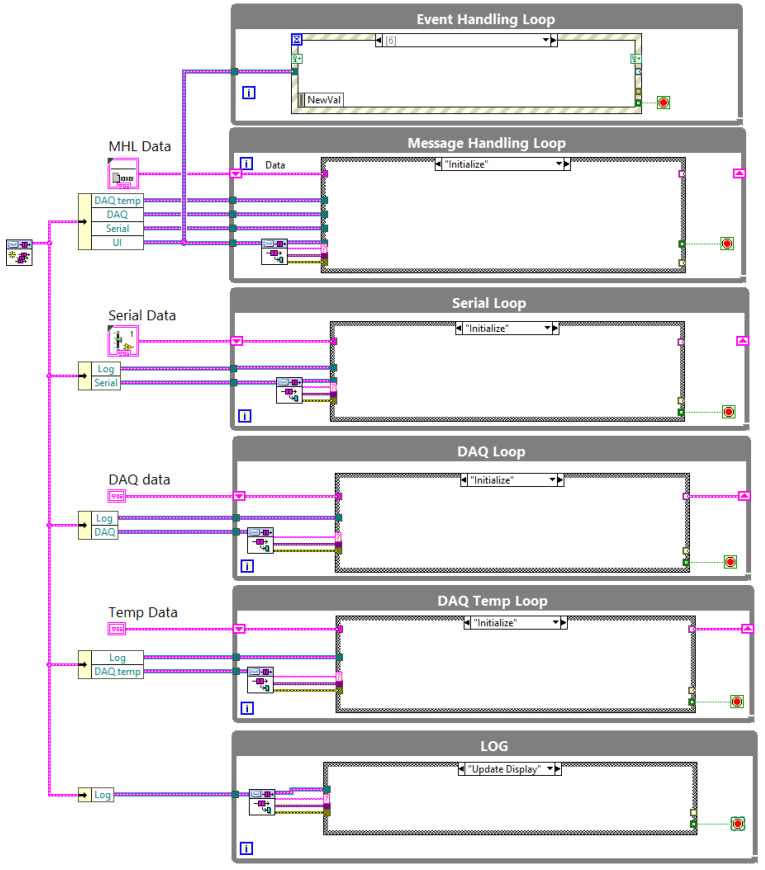
Structure of the main program.

The measurement software is written in a standard NI Queue Message Handler (QMH) template with parallel blocks of code called “drivers” whose duty is to communicate with the measurement devices and log data. The software structure is presented in Figure 4. The Event Handling Loop is used for graphical user interface (GUI) interaction, and the Message Handling Loop is the main loop which controls the measurement procedure and communicates with following loops to send commands. The next three loops (i.e., Serial Loop, DAQ Loop, and DAQ Temp Loop) communicate with the hardware devices for data acquisition. These loops communicate with the STAV 500/280 for displacement and force acquisition, cDAQ NI9216 module for resistance acquisition, and cDAQ NI9219 module for ambient temperature acquisition, respectively. The last LOG loop displays messages from the above loops to provide user feedback.

STAV 500/280 constantly generates data, so the Serial Loop works so long as the software is running. When a measurement is in progress, data are logged into a file and displayed in the front panel chart. When measurements are not in progress, the actual values of the position and force are presented in the indicator.

The measurement data are saved in TDMS format. This allows for many measurement series to be stored in one file. When the measurement procedure starts a new data group, a TDMS file is created with the proper description (date, specimen, measurement procedure, etc.). Data are logged directly into the file in real-time and not stored in RAM memory before saving files. This solution protects against data loss should a software error occur and allows for short-term measurement series to be conducted as well as long-term measurement series.

**Figure 5:**
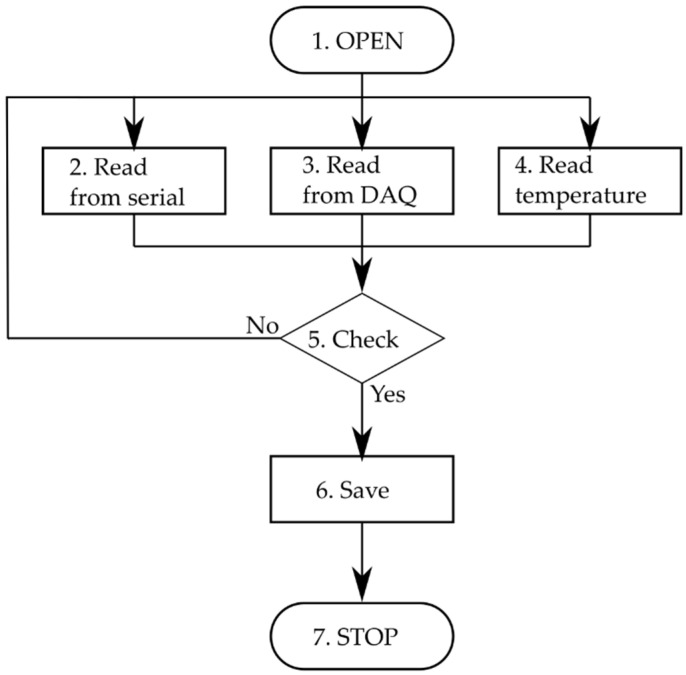
Structure of developed control algorithm.

Data synchronization is completed via timestamp using three separate loops. The data acquisition rate in Serial and DAQ loops is 10 Hz. The decision algorithm collects data as one measurement point if the delay is less than 0.04 s. Ambient temperature is recorded every 5 s. The algorithm was implemented in the Functional Global Variable (FGV) structure and is presented in Figure 5.

The first step is to open (or create) a TDMS file and is done after the RUN button is pressed. While measurements are in progress, the measurement loops activate the proper state (2, 3, or 4) with actual values. Next, the decision function (5. Check) is activated. If data are valid, the save state is activated, and the entire dataset is saved as a new measurement point. Otherwise, data are lost. The task is finished, and the file closed in Stop state once the STOP button is pressed.

## 3. Results

**Figure 6:**
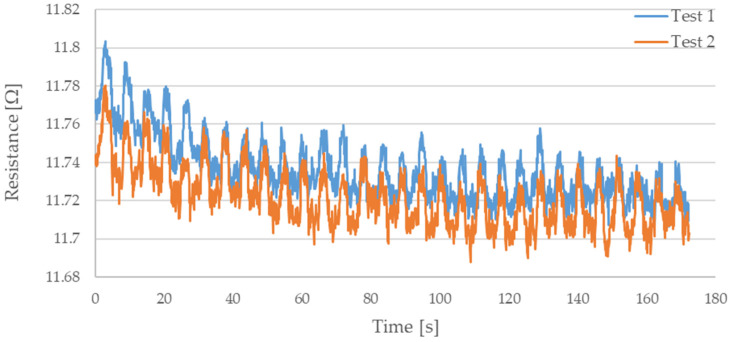
Measured resistance of Flexinol wire during cyclic stretching where strain = 0.25% (0.3 mm).

**Figure 7:**
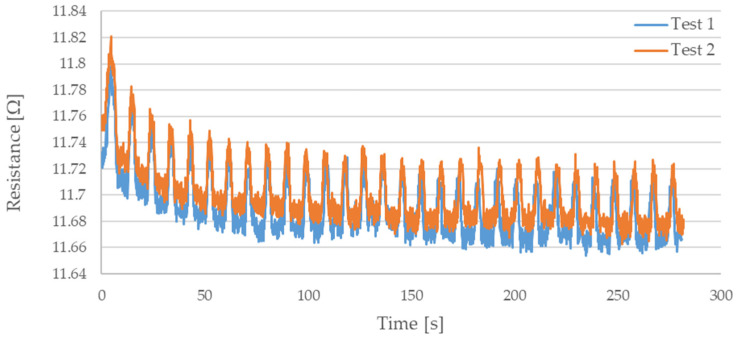
Measured resistance of Flexinol wire during cyclic stretching where strain = 0.5% (0.6 mm).

**Figure 8:**
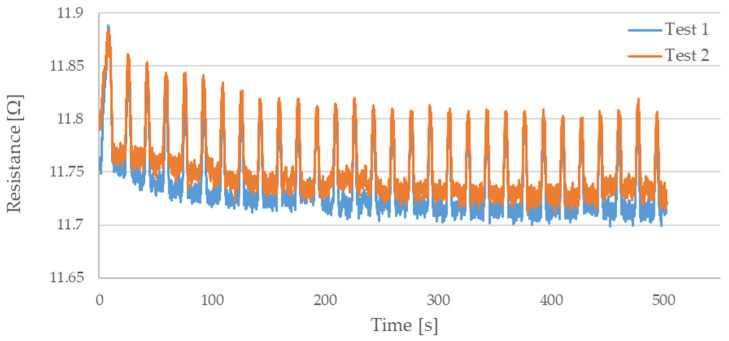
Measured resistance of Flexinol wire during cyclic stretching where strain = 1% (1.2 mm).

**Figure 9:**
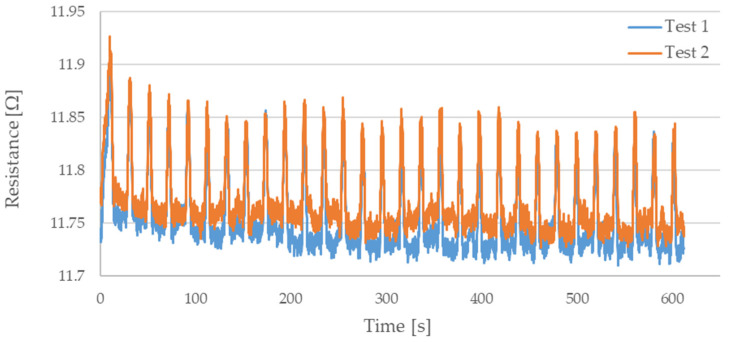
Measured resistance of Flexinol wire during cyclic stretching where strain = 1.25% (1.5 mm).

**Figure 10:**
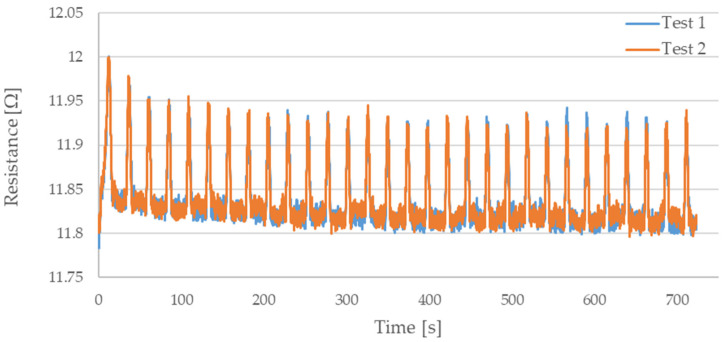
Measured resistance of Flexinol wire during cyclic stretching where strain = 1.5% (1.8 mm).

**Figure A1:**
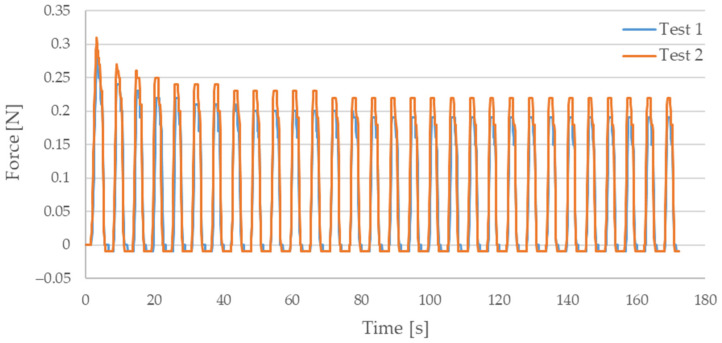
Force recorded during cyclic stretching of Flexinol wire where strain = 0.25% (0.3 mm).

**Figure A2:**
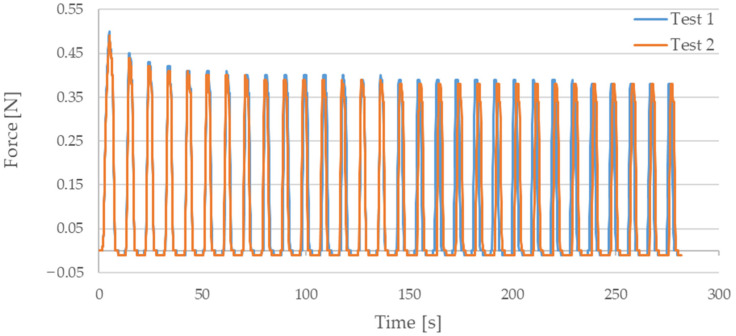
Force recorded during cyclic stretching of Flexinol wire where strain = 0.5% (0.6 mm).

**Figure A3:**
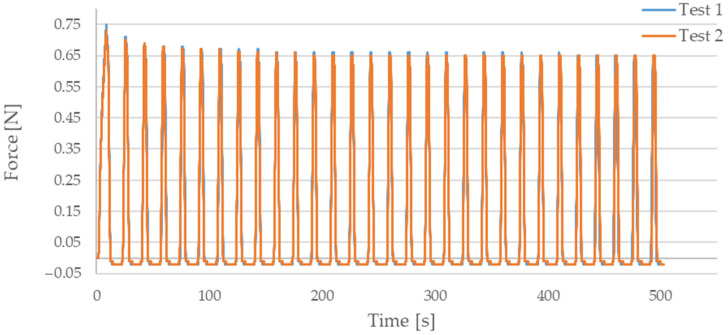
Force recorded during cyclic stretching of Flexinol wire where strain = 1% (1.2 mm).

**Figure A4:**
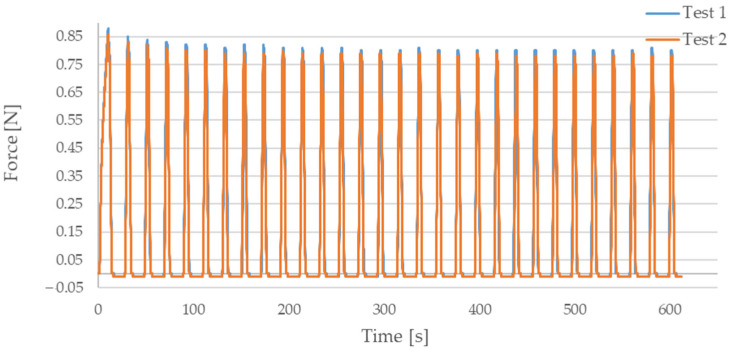
Force recorded during cyclic stretching of Flexinol wire where strain = 1.25% (1.5 mm).

**Figure A5:**
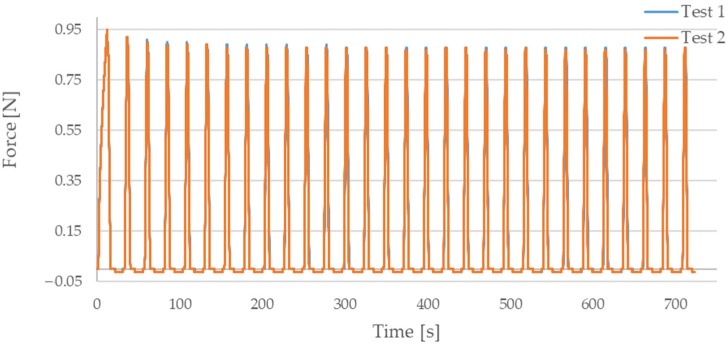
Force recorded during cyclic stretching of Flexinol wire where strain = 1.5% (1.8 mm).

During testing, the resistance of Flexinol wire under cyclic loading was measured. The force was also measured during this process. The measured resistance of the Flexinol sample during each test is presented in Figure 6, Figure 7, Figure 8, Figure 9 and Figure 10. The measured tensile force during Flexinol cyclic stretching is presented in Figure A1, Figure A2, Figure A3, Figure A4 and Figure A5 (Appendix A).

**Figure 11:**
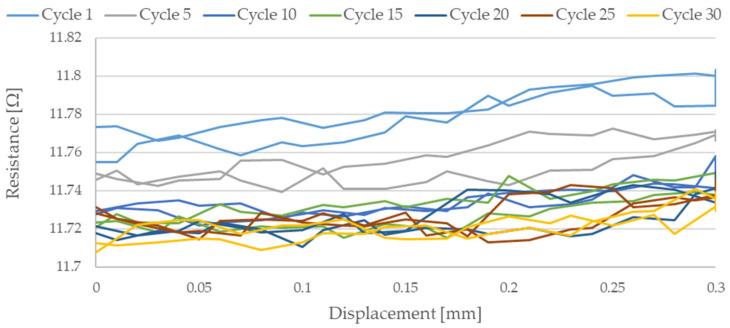
Flexinol wire resistance change during selected stretching cycle where strain = 0.25% (0.3 mm).

**Figure 12:**
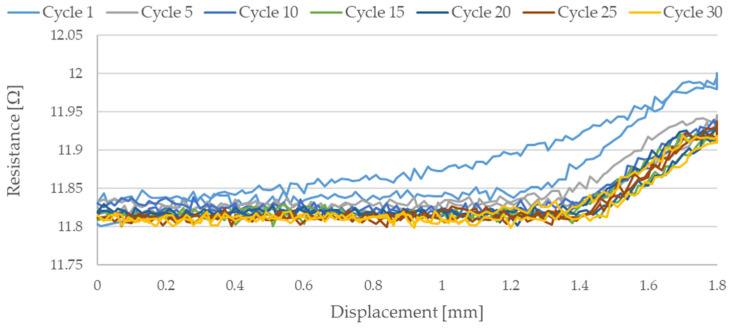
Flexinol wire resistance change during selected stretching cycle where strain = 1.5% (1.8 mm).

As shown in the graphical data illustrated in Figure 6, Figure 7, Figure 8, Figure 9 and Figure 10, the electrical resistance of the Flexinol wire changed with each stretching cycle. However, the size of this change was dependent on the assumed elongation (i.e., strain). In order to better illustrate the dependence of the change in resistance on the assumed elongation, Figure 11 and Figure 12 show the change in Flexinol resistance with respect to the assumed deformation during selected stretching cycles with 0.25% and 1.5% of elongation.

As could be seen in Figure 11, Flexinol wire resistance decreased in the presented cycles. Resistance at the beginning of each cycle is higher than at the end of the same cycle. Thus, the Flexinol resistance decreases with each successive stretch cycle. Cycle 25 is an exception, but the measurement might be disturbed at this point, which can be also seen in Figure 6. It can also be seen that the resistance decreases faster during the first 15 stretching cycles. After 15 stretching cycles, the Flexinol resistance decreased further but to a lesser extent. A similar phenomenon was observed during cycling stretching with 1.5% of assumed elongation. However, there is a difference in the first stretching cycle. In this case, Flexinol wire resistance increased after first starching cycle. This situation is also observed in the case of 1.25% of assumed elongation. In the next stretching cycles, Flexinol wire resistance decreased in each stretching cycle, as in the case of 0.25% of assumed elongation. However, after 30 stretching cycles with 1.5% of assumed elongation, the resistance of the Flexinol wire is not lower than its initial resistance. During research with smaller assumed elongation, the resistance of the Flexinol wire after 30 stretching cycles was smaller than its initial resistance.

## 4. Discussion

**Figure 13:**
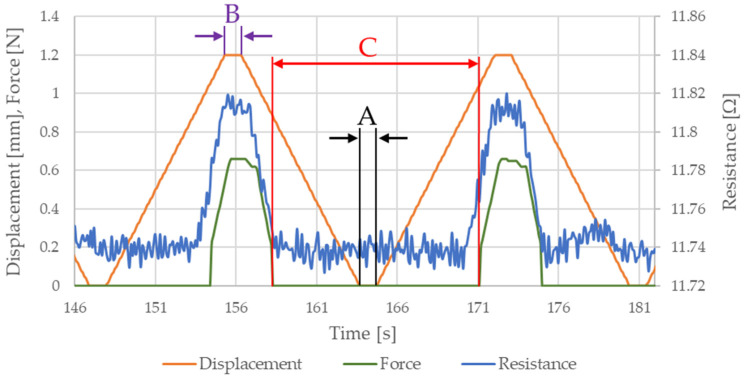
Representative data section: A—displacement = 0 mm; B—maximum displacement; C—tensile force = 0 N.

Raw data recorded during testing can be divided into three sections depending on the part of the stretching cycle. Figure 13 shows a representative section of measurement data consisting of two steps.

**Figure 14:**
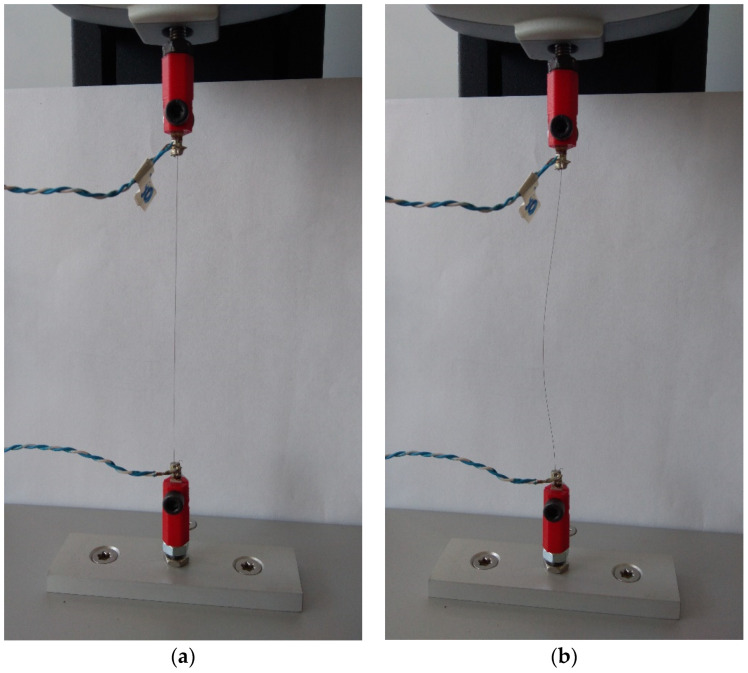
Flexinol wire mounted in testing stand: (**a**) before test; (**b**) after first stretching cycle with 1.5% strain.

Section A details the portion of the stretching cycle where displacement is 0 (i.e., initial position). Section B describes the stretching cycle where the maximum displacement (displacement value is dependent on the assumed elongation) has been achieved. Section C is the part of the stretching cycle where there is no tensile force. The length of this section is dependent on the stretching cycle number and assumed elongation since the length of the Flexinol wire changes with time (i.e., increasing length with increasing number of stretching cycles). In the first stretching cycles (when the tested material is not elongated) the stretching force begins to increase with the start of motorized vertical test stand movement. A change in Flexinol resistance occurs during the same time (when stretching force increase and motorized vertical test stand start its movement). However, after the first stretching cycle, Flexinol wire increased its length. The Flexinol wire elongation depends on the displacement assumed in the stretching cycle. Figure 14a shows the Flexinol wire sample before testing, and the wire is tight. Figure 14b shows the Flexinol wire sample following the first stretching cycle (elongation 1.5%), and the sample length has increased.

When the length of the Flexinol wire increased after the first stretching cycle, the force did not increase at the same time as the start of the motorized vertical test stand movement. The same applies to changes in Flexinol electrical resistance, because the length of the Flexinol after the first stretching cycle increased. Thus, Flexinol has to be stretched again during the motorized vertical test stand movement. After stretching (during the STAV 500/280 movement), an increase in stretching force and changes in electrical resistance are observed. That phase difference in displacement, force, and resistance depends on assumed elongation. The greater the elongation, the greater the phase difference.

**Figure 15:**
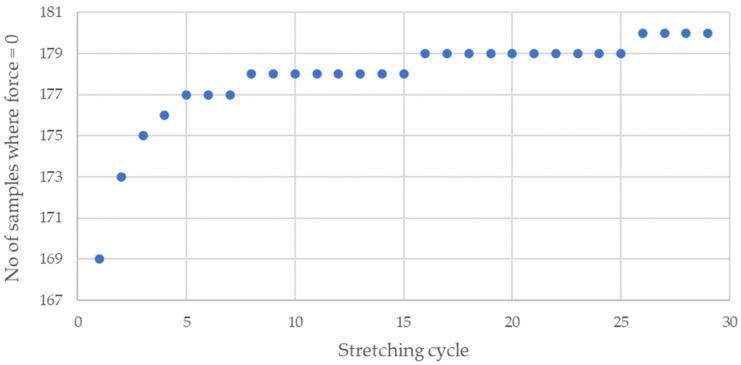
Number of data points where the force is equal to 0 [N] during Flexinol wire cyclic stretching (elongation = 1.5%).

**Figure 16:**
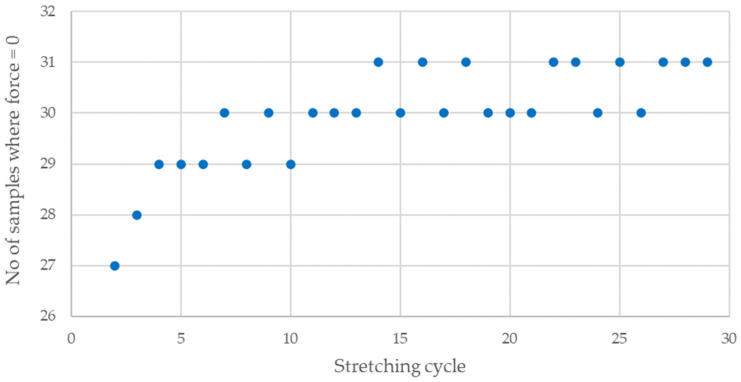
Number of data points where force is equal to 0 [N] during Flexinol wire cyclic stretching (elongation = 0.25%).

The cyclic stretching of the Flexinol wire resulted in decreased electrical resistance (Figure 6, Figure 7, Figure 8, Figure 9 and Figure 10). In all analyzed cases, a systematic decrease in the Flexinol resistance for successive stretching cycles was observed. This change was noticeable from the first cycle in the case of elongations 0.25%, 0.5%, and 1% (Figure 6, Figure 7 and Figure 8). For larger elongations of 1.25% and 1.5% (Figure 9 and Figure 10), the first stretching cycle increased the Flexinol resistance. We believe this is due to the fact that the cross-sectional area of the wire decreases when stretched above 1%. The material volume before and after deformation must be the same. Therefore, increased material length means that the diameter has decreased. This is due to permanent deformation after the first stretching cycle (Figure 14a). This is also related to the resistance. In accordance with Equation (1), decreasing cross-sectional area is accompanied by increasing resistance. The amount of elongation may be indirectly determined by counting the number of data points in raw data section C (Figure 13). The number of samples when the tensile force is equal to 0 increases when the Flexinol sample is permanently deformed (elongated) during the previous stretching cycle. The number of data points where tensile force = 0 is shown in Figure 15 (elongation = 1.5%) and Figure 16 (elongation = 0.25%).

The 0 and last steps are not calculated, because these steps correspond to the time before starting and after stopping the test. The number of data points is constant after approximately seven steps. Small distortions may by caused by air fluctuation. Distortions may be also caused due to the data recording frequency (10 Hz). For the test where the elongation was set at 0.25%, the number of data points where force was equal to 0 increased by about two during the first five cycles. However, in the test case where the elongation is set at 1.5%, the number of data points where the force is equal to 0 increased by about eight in the first five cycles. After 29 cycles, this number of data points increased by about four in the case of elongation = 0.25% and 12 for elongation = 1.5%. As such, increasing elongation increases the permanent deformation quantity. It is important to avoid this situation since it permanently increases the Flexinol resistance. For high enough elongation, the effects of Flexinol resistance decreasing during cyclic stretching could be distorted or become immeasurable. Even for small elongations, the number of data points where the tensile force is equal to 0 increased. However, the impact was much smaller on the reduction of resistance with successive stretching cycles. To avoid increasing the Flexinol resistance after the first stretching cycle, the elongation should not exceed 1%.

**Figure 17:**
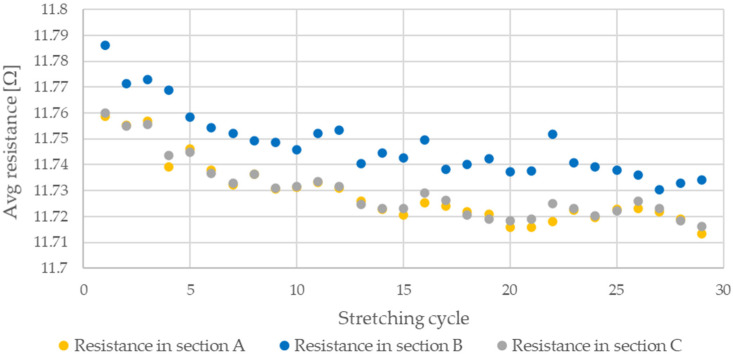
Average resistance of Flexinol wire for stretching cycle where elongation = 0.25% (0.3 mm).

**Figure 18:**
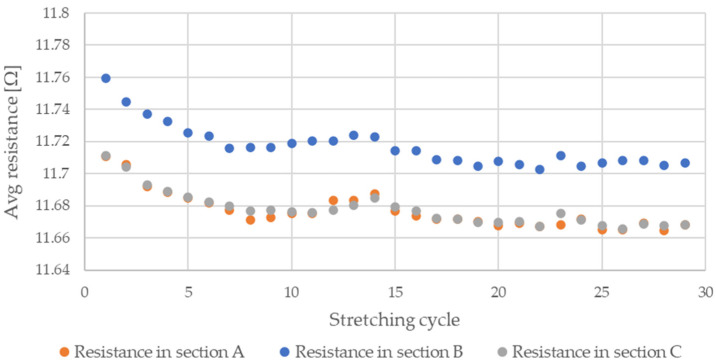
Average resistance of Flexinol wire for stretching cycle where elongation = 0.5% (0.6 mm).

**Figure 19:**
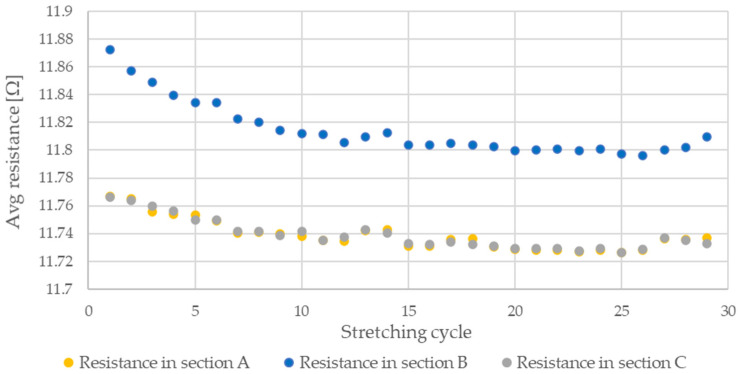
Average resistance of Flexinol wire for stretching cycle where elongation = 1% (1.2 mm).

**Figure 20:**
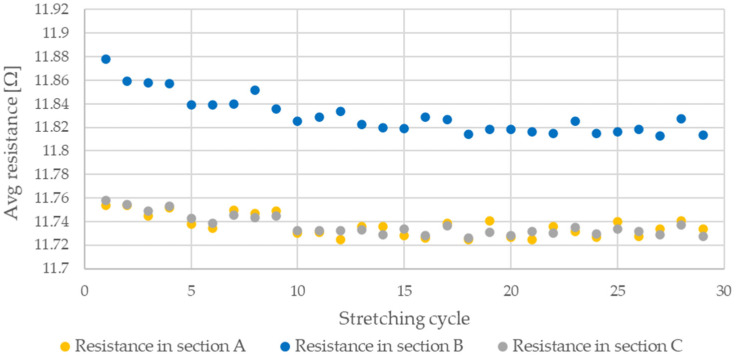
Average resistance of Flexinol wire for stretching cycle where elongation = 1.25% (1.5 mm).

**Figure 21:**
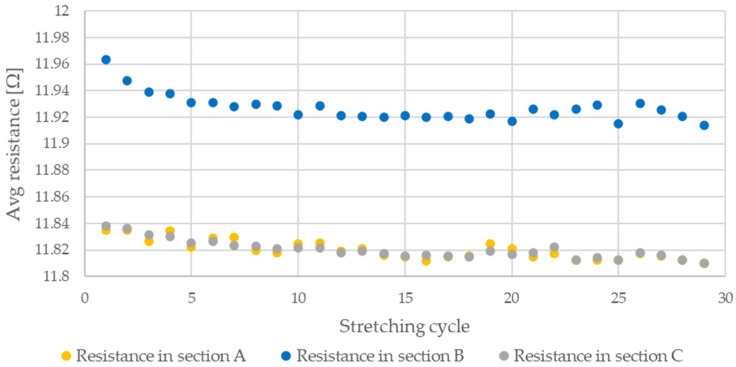
Average resistance of Flexinol wire for stretching cycle where elongation of 1.5% (1.8 mm).

The average sample resistance for sections A, B, and C (Figure 13) is presented in Figure 17, Figure 18, Figure 19, Figure 20 and Figure 21 for all analyzed elongations. The resistance of the Flexinol sample in section A is generally equal to its resistance in section C, confirming that the length of the Flexinol sample changes in a slight range.

The presented data show that the electrical resistance of the Flexinol sample decreases with each stretching cycle. This phenomenon is observed in both extreme positions. However, the stretched sample resistance is higher than the resistance of the unstretched sample. This is related to the elastic deformation of the sample that occurs during stretching (Equation (1)). However, the change in sample resistance is similar in sections A and B for each cycle. As such, changes in sample resistance for each cycle are not related to deformation. Cycling stretching with small elongation causes changes in the martensite structure (from twinned to detwinned). Increasing sample resistance during testing was observed for some points, as seen in Figure 17, Figure 18, Figure 19, Figure 20 and Figure 21. This phenomenon may be due to environmental noise factors such as air fluctuation. However, despite the outliers where sample resistance increases, the general trend shows decreasing resistance with each stretching cycle. This decrease in resistance is measurable, although very small. Resistance decrease also depends on stretching elongation. As such, the presented measurements required the use of specialized equipment capable of resistance measurements with at least 0.006 Ω accuracy. Such small changes in resistance during cycling stretching create additional requirements for the test stand configuration (i.e., to account for the influence of air and temperature fluctuations). This study is the first to examine changes in Flexinol resistance during its cyclic stretching for small elongation. Our results shows that this may be a valuable direction of further research. When Flexinol wires will be used in a passive sensor, proper calibration between displacement and electrical resistance will be important. According to the presented research, such a sensor should work within the elongation range of less than 1%. As can be seen in Figure 13, stretching force increases only when Flexinol wire is stretched (as in case of the electrical resistance). So, correct arrangement of the Flexinol wires will be also important for current applications. Change of the electrical resistance according to the elongation will also depend on the Flexinol wire length and diameter. So, when the Flexinol wire-based sensor design is finished, testing with the use of the required length and diameter of the Flexinol wire should be performed to calibrate the change of electrical resistance in accordance with the displacement. A potential application indicated by this work is the use of Flexinol wires as sensor in multilayered composites [40,41] which are widely used in many industries, such as aviation, wind turbine, and space industries [42,43]. Flexinol wire-based sensors could be used to monitor composite conditions. This type of measurement could be performed both online and offline. Proper data analysis could provide information regarding the need for a detailed inspection or replacement of a given component.

## 5. Conclusions

This research shows that the cyclic stretching for small elongations decreases Flexinol wire electrical resistance with each stretching cycle. Changes in Flexinol wire electrical resistance may be related with martensite reorientation (phase change from twinned to detwinned martensite). In the case of elongations greater than 1%, the first cycle increased the resistance. This is attributed to increasing the overall wire length. The measured resistance shifts were very small. In the first stretching cycles, resistance decreased by about 0.01–0.02 Ω during each stretching cycle. However, the more stretch cycles, the lower the change in resistance. This also depends on the applied stretching elongation. Therefore, the required accuracy of the measuring equipment should be at least 0.006 Ω. A potential application suggested by this research is the use of SMAs as passive cyclic loading sensors with a maximum load memory. Future research is currently being dedicated to the impacts of ambient temperature and strain velocity on resistance. Further research should also be focused on the sensitivity and durability of the Flexinol wire-based sensor. Further research should conducted for elongations below 1%.

## Data Availability

Data is contained within the article.

## Appendix A

The force registered by the FB5 sensor during cyclic stretching of Flexinol wire for variable elongation is presented in Figure A1, Figure A2, Figure A3, Figure A4 and Figure A5.
